# Literary Notes

**Published:** 1903-05

**Authors:** 


### LITERARY NOTES.

THE *American Journal of Dermatology and Genitourinary Diseases,* published at St. Louis, Mo., proposes to devote its entire issue for May, 1903, to a symposium on modern prostatic investigation. The leading genitourinary surgeons will participate in the discussion, which will be arranged and presented in an original manner.

---

PROVIDENCE RETREAT, a private hospital for the insane, in Buffalo, conducted by the Sisters of Charity, has issued its 36th annual report. The capacity of the institution is 125 patients, and the daily average population for the year covered by the report, ending September 30, 1902, was 119 plus. The medical officers are: William C. Krauss, medical superintendent; John J. Twohey, physician-in-charge; and W. J. O'Donnell, assistant physician.

---

NEW BOOKS.—W. B. Saunders & Company, of Philadelphia, announce the early publication of the following:

The Vermiform Appendix and its Diseases. By Howard A. Kelly, M.D., and E. Hurdon, M.D., of Baltimore.

Myomata of the Uterus. By Howard A. Kelly, M.D., Professor of Gynecology, Johns Hopkins University, Baltimore.

A Textbook of Operative Surgery. By Warren Stone Bickham, M.D., Assistant Instructor in Operative Surgery, College of Physicians and Surgeons, New York City.

The Practical Application of the Röntgen Rays in Therapeutics and Diagnosis. By William Allen Pusey, M.D., Professor of Dermatology, College of Physicians and Surgeons, Chicago; and Eugene W. Caldwell, B. S., Director of the Edward N. Gibbs Memorial *X*-ray Laboratory, and University and Bellevue Hospital Medical College, New York City.

A Textbook of Obstetrics. By J. Clarence Webster, M.D., F.R.C.P. (Edin.), Professor of Obstetrics and Gynecology, Rush Medical College, in affiliation with the University of Chicago.

A Textbook of Diseases of Women. By Barton Cooke Hirst, M.D., Professor of Obstetrics, University of Pennsylvania, Gynecologist to the Howard, the Othopedic, and the Philadelphia Hospitals.

A Textbook of Pathology. By Joseph McFarland, M.D., Professor of Pathology and Bacteriology, Medico-Chirurgical College, Philadelphia.

The Blood in its Clinical and Pathological Relations. By Alfred Stengel, M.D., Professor of Clinical Medicine, University of Pennsylvania; and C. Y. White, Jr., M.D., Instructor in Clinical Medicine, University of Pennsylvania.

A Thesaurus of Medical Words and Phrases. By Wilfred M. Barton, M.D., Assistant to Professor of Materia Medica and Therapeutics, and Lecturer on Pharmacy, Georgetown University, Washington, D. C.; and Walter A. Wells, M.D., Demonstrator of Laryngology and Rhinology, Georgetown University, Washington, D. C.

A Textbook of Modern Therapeutics. By A. A. Stevens, M.D., Lecturer on Physical Diagnosis, University of Pennsylvania ; Professor of Pathology in the Woman's Medical College, Philadelphia.

